# Special Issue “ALD Technique for Functional Coatings of Nanostructured Materials”

**DOI:** 10.3390/nano12193489

**Published:** 2022-10-05

**Authors:** Javier Garcia Fernández, Victor Vega Martínez, Victor Manuel de la Prida Pidal

**Affiliations:** 1 Departamento de Física, Facultad de Ciencias, Universidad de Oviedo, C/Federico García Lorca nº 18, 33007 Oviedo, Spain; 2 Laboratorio de Membranas Nanoporosas, Edificio de Servicios Científico Técnicos “Severo Ochoa”, Universidad de Oviedo, C/Fernando Bonguera s/n, 33006 Oviedo, Spain

Atomic layer deposition (ALD) is a vapor-phase technique that consists of the alternation of separated self-limiting surface reactions, which enable film thickness to be accurately controlled at the angstrom level, based on the former atomic layer epitaxy method [1]. The ALD technique has become an important tool for the nanofabrication of novel materials and devices at the micro–nano scale, as it can control the deposition of pin-hole-free thin films with an atomic-level thickness and composition across a wide variety of high-aspect-ratio surfaces [2,3]. In this regard, several approaches based on ALD processes can be used to tune both the surface chemistry and geometry of different types of functional membranes and nanoporous patterned templates [4,5]. The ultra-precise thickness control of thin layers and 3D conformability of the surface coatings of materials achieved through the ALD technique have driven the development of new material structures and novel devices with improved efficiency. These have been employed in different research fields, such as magnetism and spintronics [6,7,8,9], catalysis [10], biochemistry [11], photonics [12], clean and sustainable energy conversion and renewable energy storage [13,14,15], among others. Recent investigations in ALD research are still focusing on the development of novel materials and their growth mechanisms, and designing new precursors and deposition approaches. Moreover, the implementation of such techniques in combination with existing technologies will give rise to new downsized devices with superior a performance and durability. In addition, the ALD process is required in many research areas where the surface protection of nanostructures is urgently required, allowing for their manipulation and characterization in order to advance fundamental research and quantum phenomena investigations.

This Special Issue summarizes the most recent studies highlighting the synthesis, characterization, and applications of novel nanomaterials fabricated through the ALD technique. These studies explore new materials, precursor gases and several strategies and approaches for depositing novel nanomaterials in different applications across a great variety of research fields, such as energy conversion and storage, spintronics, photonic devices and biochemistry. This issue comprises a total of seven contributions [16,17,18,19,20,21,22]. The wide variety of applications covered by these seven articles demonstrates the increased attention that the ALD technique has recently received. In this regard, the excellent study by Tsai et al. [16] introduces a brief review of the main advantages and shortcomings of the recent advances made in spintronic applications by means of atomic layer technologies, including atomic layer etching (ALE) and ALD techniques. First, they introduce the main atomic layer deposition techniques and review the current developments in both atomic layer technologies for spintronic applications. Next, they focus on the main aspects of ALD technique. Then, the ALE technology for insulators, semiconductors, metals, as well as newly designed two-dimensional van der Waals materials are briefly discussed. Currently, these two deposition methods are essential for the fabrication of chips and sensors, where the materials involved are quite complex, particularly those associated with composite materials, such as alloys and heterojunctions. Additionally, a comparison between the critical factors of ALD for constructing ALE technology is explored in depth, and finally, the authors discuss the future prospects and challenges of both atomic layer technologies in the specialized field of spintronics.

The fundamental research conducted for the development of new nanostructured materials and the discovery of novel routes for deposition by the ALD technique are the main topics covered by the articles collated in this Special Issue. Indeed, the article by Austin et al. [17] discusses the successful design and fabrication of a novel, high-temperature (over 800 °C) ALD reactor for the growth of 1D nanostructured GaN thin films and 3D conformal coatings on silica nanosprings by employing different buffer layers of AlN and Al_2_O_3_/AlN. Contrastingly, Waleczek et al. [18] focus their research on the development of a novel low-temperature (under 100 °C) ALD deposition method to introduce alumina into TiO_2_ thin films and inverse opal photonic crystal nanostructures in order to study the influence on their thermal stability and optical properties, showing a clear enhancement in both. Furthermore, in the study carried out by Zhang et al. [19], the authors explore the optimal conditions for oxygen plasma power to obtain high-quality SiO_2_ thin films fabricated by a remote plasma atomic layer deposition (RP-ALD) technique, which plays an important role in the surface oxidation reaction. They also study the influence of annealing temperature in later thermal treatments on the increase in refractive index due to a higher density. Overall, the most effective combination of both strategies would be ideal for its application in semiconductor-related industries in the near future.

Integrated flexible electrodes are of great importance for many applications, including electrochemical energy storage, electrocatalysis and solid-state memory devices. Indeed, the communication by Yun et al. [20] focuses on the design and fabrication of flexible 3D nanostructured electrodes, consisting of highly ordered arrays of TiN nanotubes grown by ALD on a patterned nanoporous alumina template and deposited on a Pt substrate through and intermediate Ti layer. They proved that the optimal adhesion, mechanical and flexibility properties of TiN nanotube arrays deposited on the Pt substrate are strongly improved by employing a Ti interlayer that avoids any delamination and faceted phenomena. At the same time, I-V curves also demonstrate the good electrical contact exhibited by TiN nanotube arrays deposited on the Pt substrate, confirming their feasibility as electrodes with outstanding features for their implementation in flexible electronic devices.

Finally, the last two articles included in this Special Issue report on the antimicrobial and bactericide applications of nanostructured semiconducting materials deposited by ALD. The study by de Castillo et al. [21] addresses the antimicrobial properties exhibited by ZnO nanotubes grown by the ALD process over electrospun polyvinyl alcohol nanofibers used as polymeric substrates, in order to obtain an antimicrobial polymeric bilayered nanostructured material with a high specific surface area. García et al. [22] performed a detailed study on the influence of the conformal coatings of a patterned surface of food-grade 316L stainless steel with TiO_2_ thin layers covered by ALD, together with the addition of Ag nanoparticles by electroless plating, in order to reduce the bio-fouling produced in pipelines and buckets typically employed in dairy and agri-food industries.

As guest editors of this Special Issue, we sincerely acknowledge the significant effort and care demonstrated by all the authors who have contributed their latest research on this particular topic to this Special Issue. At the same time, we hope that the outstanding scientific quality of these selected studies—which focus on novel approaches to deposition processes, new fabrication strategies, nanostructured materials with improved physico-chemical properties, and the development of novel devices with high-performance applications related to the ALD technique—fully engages the interest of readers.
